# What Affects the Diffusion of New Energy Vehicles Financial Subsidy Policy? Evidence from Chinese Cities

**DOI:** 10.3390/ijerph17030726

**Published:** 2020-01-22

**Authors:** Weixing Liu, Hongtao Yi

**Affiliations:** 1 School of Public Administration and Policy, Renmin University of China, Beijing 100872, China; liuweixing@ruc.edu.cn; 2 John Glenn College of Public Affairs, The Ohio State University, Columbus, OH 43210, USA

**Keywords:** new energy vehicles, policy diffusion, local financial subsidy policy, event history analysis

## Abstract

Designing and implementing effective new energy vehicle (NEV) policy are policy priorities for policymakers and energy policy scholars. However, the formulation, adoption, and diffusion of the NEV policies have not been fully examined in the extant literature. This article explores the mechanisms driving the diffusion of local financial subsidy policy for NEVs in China. In this context, we aim at analyzing the factors affecting the diffusion of local financial subsidies for NEVs in cities, to explain why some cities have taken the lead in adopting local financial subsidy policies for NEVs, while other cities have lagged behind. Based on a data set of 286 cities in China from 2009 to 2016, and with event history analysis (EHA) to analyze the strategic behaviors of local governments, we found that the number of the city’s neighbors that have adopted the NEV policy, the financial incentive policy of the provincial government, the administrative ranking of the city, the city’s financial situation and innovation capacity have a direct impact on whether the city adopts a local financial subsidy policy for NEVs. This study has practical implications for policymakers in designing and promoting the spread of NEV policies.

## 1. Introduction

Countries all over the world face urgent challenges from climate change and environmental pollution [1,2]. It is a scientific agreement that greenhouse gases (GHGs) and harmful substances resulting primarily from the combustion of fossil fuels have contributed to climate change and serious environmental pollution [3,4]. As a response, countries are developing green technologies and promoting the use of renewable energy sources, including new energy vehicles (NEVs), gas turbines, wind energy, solar energy, and modern biomass energy, to reduce their dependence on fossil fuels [5,6,7]. Among these climate mitigation approaches, the development of NEVs is considered an important strategy as it targets the transportation sector, which accounts for 24.7% of total carbon emissions in the world. NEVs are defined as vehicles that primarily or exclusively use new energy with innovative power systems, including pure electric vehicles, plug-in hybrid electric vehicles, fuel cell vehicles, and hydrogen-based fuel-cell cars [8,9,10]. Many countries are vigorously developing and promoting the deployment of NEVs due to the prominent role of NEVs in reducing vehicle emissions, such as CO, SO_2_, and NOx [11,12,13]. Over the last couple of years, the United States (U.S.) government has invested billions of dollars in supporting NEVs. In 2009, the U.S. federal government enacted tax credits to increase consumer demand for plug-in hybrid electric vehicles [14]. The Japanese government has also provided support for developing vehicles with alternative powertrains to reduce emissions and oil-dependence since the early 1970s [15]. Battery-powered electric vehicles were chosen as the most promising option for the future [15,16]. The “Next-Generation Vehicle Plan 2010” set the government targets of the sale’s share of ordinary hybrid vehicles (HVs) at 20%–30%, electric vehicles (EVs) and plug-in hybrid electric vehicles (PHEVs) at 15%–20% in Japan [16]. The European Union (EU) has also adopted measures, such as incentives and compulsory measures, to promote the development of NEVs. Since 2009, a mandatory CO_2_ standard has been applied to new passenger cars and light commercial vehicles in the EU [17]. And the EU’s TEN-T program invested more than 4 million Euros of funding in 155 fast-charging stations along the main motorways in northern Europe from 2007 to 2013 [18]. Many EU countries, including Germany, the United Kingdom, France, and the Netherlands, formulated fiscal incentives to incentivize the deployment of EVs, to stimulate investments in charging infrastructure, and to support national EVs technology innovation [17]. Among them, the German government planned to achieve the target of having one million EVs on the roads by 2020 [19]. The United Kingdom and France announced that they will ban the sale of fossil fuel-powered cars in their own countries by 2040 [20].

There exists a rich literature on the technological, policy, and economic aspects related to the development of NEVs. Studies explored how NEVs can reduce GHGs emissions and alleviate environmental pollutions [3,4,11,16,21]. Other studies have focused on the diffusion of NEV market through studying the influence of consumer preferences, and psychological and socio-demographic factors [22,23,24]. There is also a stream of comparative studies on government policies and the development of NEVs across different countries. For example, Tietge et al. investigated incentives for electric vehicles in the five largest EV markets in Europe, including Germany, the United Kingdom, France, the Netherlands, and Norway [17]. Helveston et al. compared whether subsidies stimulated electric vehicle adoption in the U.S. and China [22]. In addition, many studies explored the influence of government subsidies and support policies on NEVs [7,15,25,26,27]. Quite several studies focused on predicting the future development prospects of NEVs [21,28].

However, some areas of the studies warrant further research. First, only a small number of studies explore NEVs from a micro-level perspective to examine city-level policies and incentives. Second, most studies are focused on NEVs themselves, and their impact, as well as the impact of government policies on NEVs. Few studies focus on the driving factors that influence the adoption of government policy for NEVs. Little is known about why and how the financial subsidy policy of NEVs is formulated, adopted, diffused, and implemented. This article attempts to fill the gaps in the literature on NEVs by examining the determinants of the diffusion of the financial subsidy policy of NEVs among 286 cities in China, through event history analysis (EHA).

The following section presents the background of the financial subsidy policy of NEVs in China. Then, based on a review of literature on policy diffusion and NEVs, we articulate why and how regional diffusion, policy incentives and guidance mechanisms of superior government, city’s administrative ranking, financial dependence, innovation capacity, and environmental pollution of a city affect the adoption of financial subsidy policy for NEVs. Next, we discuss the EHA method and the collection of data. We then analyze the influencing factors for the adoption of a financial subsidy policy for NEVs with EHA. We discuss the empirical results in terms of their theoretical and empirical implications.

## 2. The Financial Subsidy Policy for NEVs in China

In recent decades, the Chinese economy has witnessed fast and sustained growth. However, rapid economic development has also caused severe challenges in energy security [29,30] and environmental pollution [4,31,32]. To fundamentally transform the fossil fuel-based energy consumption structure and to reduce environmental pollution and dependence on oil imports, the Chinese government adopted the strategy to support the development of NEVs [6,20,26]. The Chinese National Development and Reform Commission (NDRC) issued the “Manufacturing Access Management Rules for NEVs” ([2007] No. 72) on 17 October 2007, which established preliminary guidelines for China’s NEV industry. To promote the technological progress of NEVs, and to encourage manufacturing companies to develop and produce NEVs, the Chinese central government has improved the initial rules with additional regulations and policy incentives for NEVs. On 17 June 2009, the Chinese Ministry of Industry and Information Technology issued the “Manufacturing Companies and Product Access Management Rules for NEVs” ([2009] No. 44). These rules regulate the production of NEVs and companies. On 28 June 2012, the Chinese central government formulated the “Energy-saving and NEV Development Plan (2012–2020)” ([2012] No. 22). It mentioned that the accumulative productions and sales volume of NEVs would be projected to be more than 5 million NEVs by 2020.

The central government’s financial subsidy policies for NEVs started in 2009. The Chinese Ministry of Finance and the Chinese Ministry of Science and Technology jointly formulated the “Notice on Pilot Work of Energy-saving and NEV Demonstration and Promotion” ([2009] No. 6) on 23 January 2009. Thirteen cities were selected as pilot areas to demonstrate and deploy NEVs, including Beijing, Shanghai, Chongqing, Changchun, Dalian, Hangzhou, Jinan, Wuhan, Shenzhen, Hefei, Changsha, Kunming, and Nanchang. The Chinese central government began to provide financial subsidies for NEVs in these pilot cities in public transportation, such as bus, taxi, logistic, and sanitation vehicles, and for postal services. The highest subsidies were offered at 60,000 RMB Yuan for pure electric vehicles [6]. Some local governments among these thirteen cities, as well as other local governments, such as Foshan, Zhuhai, Zhengzhou, have also formulated local financial subsidy policies to support the purchase of NEVs and the construction and maintenance of supporting infrastructure. 

To further promote the development of NEVs in China, the Chinese Ministry of Finance, the Chinese Ministry of Science and Technology, the Chinese Ministry of Industry and Information Technology, and NDRC jointly issued the “Notice on Pilot Subsidies for Private Purchase NEVs” ([2010] No. 41) on 31 May 2010, which extended financial subsidies for public transportation to private-owned NEVs, and selected five cities of Shanghai, Changchun, Shenzhen, Hangzhou, and Hefei as pilot cities. In 2013, The Chinese central government enlarged the pilot group to incorporate 88 cities. Since then, the central government has introduced other policy incentives for NEVs.

**Figure 1:**
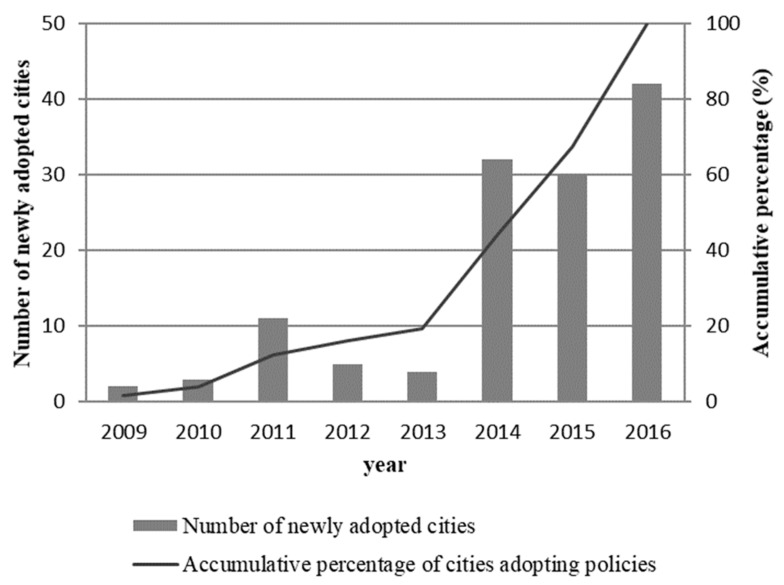
Diffusion of the financial subsidy policy of new energy vehicles (NEVs) in China.

Encouraged by the central government’s policies, many provincial and municipal governments also started to use financial instruments to subsidize NEV manufacturing companies and purchasers. Table 1 shows the number of cities that recently adopted financial subsidy policy for NEVs. And Figure 1 shows the diffusion of the local financial subsidy policies for NEVs in China from 2009 to 2016. Only two cities adopted a local financial subsidies policy for NEVs in 2009. Since then, the number of cities adopting policies has increased steadily. Eleven new cities adopted financial subsidy policies in 2011. Since 2014, no fewer than 30 cities adopted the policy every year. This illustrates a rapid diffusion pattern among local governments in the adoption of a financial subsidy policy for NEVs.

**Figure 2:**
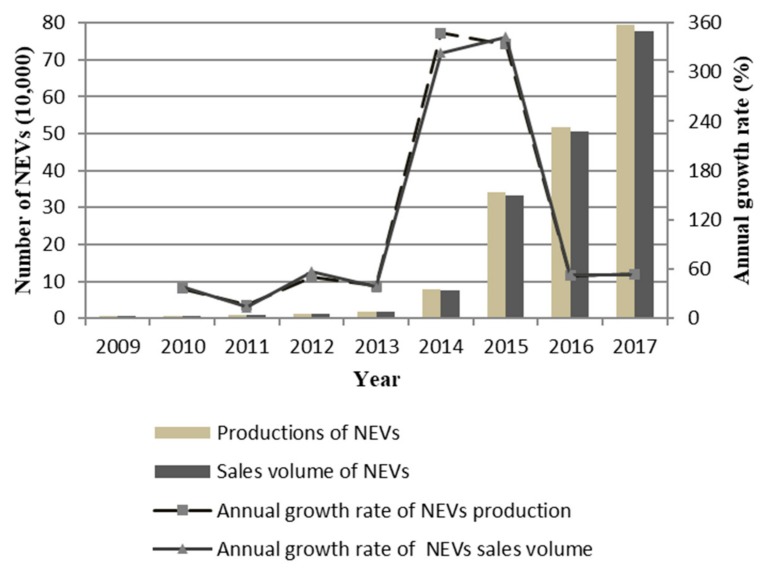
Productions, sales volume, and annual growth rate of NEVs between 2009 and 2017 in China.

With the support of government policies, the productions and sales volume of NEVs in China is also growing rapidly. Figure 2 shows the productions, sales volume, and annual growth rate of NEVs in China from 2009 to 2017, the data for which were obtained from the official website of the China Association of Automobile Manufacturers.

From 2009 to 2011, the annual production and sales volume growth rate of NEVs maintained a high level, but the total production and sales volume were not sufficiently high. This may be due to consumers’ low acceptance level and relatively high prices of NEVs. It was not long until the NEV policy incentive policies began to show substantive effects. China’s NEVs productions and sales volume exceeded 10,000 in 2012, reaching 12,552 and 12,791, respectively. The productions and sales volume of NEVs in 2014 and 2015 soared, with growth rates higher than 320%. Over the next two years, the annual growth rate stabilized at more than 50%. The productions of NEVs increased from 345,500 in 2015 to 517,000 in 2016 and reached 794,000 in 2017. The change in sales volume of NEVs is similar to that of production. Under the stimulation of government policy, especially the financial subsidy policy, China’s NEVs have made great progress in the nine years from 2009 to 2017. The production and sales volume of NEVs increased by 148 times.

## 3. Theoretical Frameworks and Hypotheses

In this section, we explore what factors affect the diffusion of the local financial subsidy policies for NEVs among cities based on current literature on policy diffusion and NEVs. Policy diffusion usually occurs when decisions of governments are influenced by earlier decisions of other governments [33]. Academia has widely studied the policy diffusion mechanism since the early works [34,35,36]. Regional diffusion is a classical and frequently mentioned mechanism of policy diffusion [36]. Regional diffusion captures the mechanism of horizontal diffusion, for which most studies focus on the influence of policy adoptions by next door or nearby geographic neighbors in prompting a government to adopt the same policy [34,36,37]. According to the financial subsidy policies for NEVs of the Chinese central government, when a local government adopts local financial subsidy policy for NEVs, it can receive matching subsidy funds from the central government, which will help develop local NEV industry and expand the market of NEVs, thereby promoting local economic development. Therefore, if a local government has adopted local financial subsidies policy, its neighboring local governments will also attempt to adopt the policy to promote local development. Based on the above argumentation, we propose the following hypothesis.

**Regional** **Diffusion** **Hypothesis** **(H1):** *The higher the percentage of neighboring cities that adopted local financial subsidy policies for NEVs in the same province, the more likely the city is to adopt local financial subsidy policy for NEVs.*

Current literature indicates that top-down coercion dynamic is also an important mechanism affecting policy diffusion [38,39,40]. It refers to conditions in which policy decisions of a government are influenced by coercive power from higher-level governments [38]. For example, local governments will accelerate the adoption of state policies under strong financial incentives of the federal government [36,38,41]. Although China’s economic system has gradually shifted from a centrally planned economy to a market economy since the Economic Reform and Opening-Up in 1978, the administrative management system continues its top-down command-and-control model [42]. The Chinese central and provincial governments frequently support or terminate local policy innovation through various policy tools, such as coordination, guidance, protection, incentive, and even political punishment [43,44,45]. Therefore, the policy adoption of local governments in China will be affected by the strategic behaviors of higher-level governments. The local financial subsidy policy for NEVs is a pilot policy that has not been implemented nationwide. Therefore, decision-makers of local governments are more influenced by the behaviors of the central and provincial governments when deciding whether to adopt it or not.

**Top-Down** **Authoritarianism** **Hypothesis** **(H2a):** *A city is more likely to adopt a local financial subsidy policy for NEVs if the provincial government the city is embedded in has adopted financial subsidy policy.*

**Top-Down** **Authoritarianism** **Hypothesis** **(H2b):** *A city is more likely to adopt local financial subsidy policy for NEVs if the provincial government the city is embedded in has developed an informal leading group or held joint meetings for NEVs.*

As discussed above, under the Chinese authoritarian administrative system, lower levels of government must follow commands and policies of higher levels of government, because most of the local government officials were appointed by the communist party committee and government at the higher level, instead of being elected by citizens [46]. In particular, the government with higher administrative ranking has more contacts with central and provincial governments and usually receives more attention from them. As a result, these cities are more likely to be coerced. Therefore, they usually respond more actively and follow the policies of the central and provincial governments.

**Administrative** **Ranking** **Hypothesis** **(H3):** *The higher the level of a city’s administrative ranking, the more likely the city is to adopt local financial subsidy policy for NEVs.*

The government’s financial situation is also an important factor affecting policy innovation and diffusion. The short-term fiscal health of a local government is an important determinant of the motivation of the government to adopt innovative policies [36], especially those policies that have an impact on the financial health of local governments. Policy innovation in one functional area is moderately related to spending by governments for that function [47]. Local governments that adopted a financial subsidy policy of NEVs need to use their own public financial resource to subsidize the purchasers of NEVs, which will put heavy pressure on local governments’ public finance. Therefore, we argue that local governments with a good financial situation will be able to adopt a financial subsidy policy for NEVs. But those with a poor financial situation may be less likely to adopt it.

**Financial** **Dependence** **Hypothesis** **(H4):** *The better the financial situation of a city, the more likely the city is to adopt local financial subsidy policy for NEVs.*

The innovation capacity of cities is also a factor influencing the adoption and diffusion of policies. Innovation capacity is the ability of a jurisdiction to produce a flow of new technologies over the long term. This new technology also includes the formulation or adoption of new policies. The innovation capacity of a country or a city depends on the existence of strong common innovation infrastructures, such as the environment and mechanisms to support innovation, and the accumulative “stock” of technological knowledge [48]. Therefore, cities with strong innovation capacity are more likely to learn from other governments and adopt new policies.

**Innovation** **Capacity** **Hypothesis** **(H5):** *The stronger the innovation capacity of a city, the more likely the city is to adopt local financial subsidy policy for NEVs.*

As mentioned earlier, reducing environmental pollution is an important reason for the development of NEVs in many countries. Several studies have also confirmed that the development of NEVs can bring about environmental benefits, such as reducing greenhouse gas emissions and improving air pollution [4,11,16,21]. The policies that produce environmental benefits may be especially attractive to local governments with serious environmental pollution [49]. Therefore, we propose the following hypothesis.

**Environmental** **Pollution** **Hypothesis** **(H6):** *The more serious a city’s environmental pollution, the more likely the city is to adopt local financial subsidy policy for NEVs.*

## 4. Method and Data

### 4.1. Model

We tested the hypotheses on the diffusion of local financial subsidy policy for NEVs in Chinese cities through EHA models. Scholars commonly use EHA to study policy diffusion, after it was first used in the classical Berry and Berry (1990) state lottery adoption study. EHA is able to explain and model diffusions of the political behavior of individuals, and rules and policies of organizations or governments, and is thus considered an ideal methodology for the study of policy diffusion [36,50]. The most frequently used EHA model has three alternative link functions, including Logit, Probit, and Cloglog [51]. Following the EHA with a logistic regression approach in diffusion studies in China [33,46], we also employed EHA with logit models, as it is easier to interpret the coefficients as odds ratios in logit [46]. The EHA model assumed that the size of the risk set will decrease (when the event in question cannot be repeated by an individual, e.g., death) or stable (when the event in question can be repeated by an individual, e.g., moving one’s residence) over time as individuals in the sample experience the event [36]. When observations are temporally related, the results of a logit analysis may be misleading [52]. Therefore, we resort to a simple remedy of binary time-series-cross-section (BTSCS) in our EHA model [53,54]. The BTSCS can help us create temporal dummies for each period and then use cubic spline smoothing functions to smooth the temporal dummies. Three equally spaced natural cubic splines will be produced and then incorporated into the model [53]. In this way, temporal dependence can be tested and controlled.

### 4.2. Dependent Variable

This article takes the adoption of local financial subsidy policy as our dependent variable. According to the tradition of EHA, we operationalize the dependent variable as a binary variable. If a city adopted the local financial subsidy policy in year t, then the dependent variable is coded as “1”, otherwise, it is coded as “0”. And observations after year t are removed according to the requirement of the EHA method. We observed 293 cities of prefecture level and above in China from 2009 to 2016. A few cities were dropped from observations due to a lack of statistical data for some years. For example, Chaohu City was abolished in 2011. Moreover, Bijie City, Tongren City, Sansha City, Haidong City, Danzhou City, and Turpan City were formally established after 2010. Their socio-economic data are incomplete and unavailable in China’s City Statistical Yearbook. Therefore, we excluded the observations of these seven cities, and the final number of cities was 286. The dependent variable covers 286 cities from 2009 to 2016. The mean value of the dependent variable “Adoption” was 0.062.

Note that we also spent much time collecting and sorting out the local level subsidy value provided by each city. However, many cities have not formulated concrete subsidy standards. Although some cities have subsidy standards, many subsidies are specified according to different vehicle models, power types, driving mileage, battery capacity, etc. Therefore, it is difficult to describe and summarize the value of subsidies offered at the local level. In other words, we do not have subsidy value data for the majority of cities in our study sample. Therefore, we are not able to estimate models with continuous values of the subsidies, even though we agree it would be a more accurate measure than the simple dummy variable operationalization. Measures and data sources of the variables are shown in Table 2. The descriptive statistics of these variables are presented in Table 3.

### 4.3. Independent Variables

In the previous section, we proposed six hypotheses on how regional diffusion, top-down influences of policy incentives and guidance mechanism of superior government, administrative ranking, financial dependence, innovation capacity, and environmental pollution of Chinese local governments affect the adoption of local financial subsidy policy for NEVs.

First, we treated the accumulated percentage of city governments that had adopted local financial subsidy policy for NEVs within each province by the same year as our independent variable “Regional Diffusion”, which we used to test the regional diffusion hypothesis. In our observation samples, the maximum value of the “Regional Diffusion” was 92.31%, the mean value was 10.61%, and the minimum was 0, which denotes that no city in this province adopted local financial subsidy policy for NEVs.

Then we included two variables, named “Policy_Sup_Gov” (policy incentive of superior government) and “Guidance_ Sup_Gov” (guidance mechanism of superior government) to test the top-down authoritarianism hypothesis. We operationalized the two variables as binary variables. If a provincial government first issued a financial subsidy policy in year t, then the variable “Policy_Sup_Gov” was coded as “1” from year t to 2016. Otherwise, it was coded as “0”. The coding of variable “Guidance_ Sup_Gov” which denoted whether the provincial government established a leading group or contact meeting system for promotion and application of NEVs was the same as the variable “Policy_Sup_Gov”. The mean values of “Policy_Sup_Gov” and “Guidance_ Sup_Gov” were close, which were 0.249 and 0.269, respectively.

The fourth independent variable was “Administrative Ranking”, which gauged the administrative ranking of a city. According to the division of the city’s administrative ranking in China, we divided it into four categories. In our 286 sampled cities, there were four centrally-administered municipalities, which were coded as “3”, fifteen sub-provincial-level cities coded as “2”, seventeen provincial capitals but not sub-provincial-level city coded as “1”, and the remaining normal prefecture-level cities were coded as “0”. 

The independent variable “Financial Dependence” was used to measure the financial situation of a city government. It is calculated as:

(1) $${\mathrm{Financial}\;\mathrm{Dependence}}_{i,\;t}=\;\;\frac{\;\mathrm{expenditure}\;\mathrm{of}\;\mathrm{city}\;i\;\mathrm{in}\;\mathrm{year}\;t-\;\mathrm{income}\;\mathrm{of}\;\mathrm{city}\;i\;\mathrm{in}\;\mathrm{year}\;t}{\;\mathrm{expenditure}\;\mathrm{of}\;\mathrm{city}\;i\;\mathrm{in}\;\mathrm{year}\;t}\;\times \;100\%.$$

The public finance expenditure and income were measured in the unit of 10,000 RMB Yuan. The value of “Financial Dependence” reflected the financial situation of the city government. A smaller value indicates lower financial dependence of the city government on the superior government, and thus a better financial situation. In our observation samples, the maximum value of the “Financial Dependence” was 95.4 which indicates that 95.4% of this city’s public finance expenditure could not be supported by its public finance income, and the minimum was −11.6, which means that its public finance income could fully meet public finance expenditure and had a surplus. The mean was 54.376.

We tested the innovation capacity hypothesis by including the independent variable “Innovation Capacity”. It is calculated as:

(2) $${\mathrm{Innovation}\;\mathrm{Capacity}}_{i,t}=\frac{\mathrm{quantity}\;\mathrm{of}\;\mathrm{invention}\;\mathrm{patent}\;\mathrm{grant}\;\mathrm{of}\;\mathrm{city}\;i\;\mathrm{in}\;\mathrm{year}\;t}{\mathrm{total}\;\mathrm{household}\;\mathrm{registration}\;\mathrm{population}\;\mathrm{of}\;\mathrm{city}\;i\;\mathrm{in}\;\mathrm{year}\;t}.$$

The total household registration population of city was measured in the unit of 10,000 persons. In our observation data, the maximum value of this variable was 34.859, indicating that the city had a strong innovation capacity, and the minimum value was 0, which means that the innovation capacity of the city was poor.

The sixth independent variable was “Environmental Pollution”. We measured environmental pollution by the volume of SO_2_ emissions in each city in the unit of tons. The higher the volume of SO_2_ emissions, the more severe the environmental pollution of the city. The maximum value of “Environmental Pollution” was 586,000. However, the minimum was 0.47. This indicates that the environmental pollution of different cities in the observation period was quite different.

### 4.4. Control Variables

Taking into account the impact of social, economic, and natural conditions on the adoption of local financial subsidy policy for NEVs, we included a set of control variables in the analysis. GDP (gross domestic product) growth rate, GDP per capita, and Secondary Industry (proportion of secondary industry) measure the state of economic development and wealth in the city. The control variables population, City Road Area, Public_Transp_Vs (public transportation vehicles per 10,000 persons), Taxis (taxis per 10,000 persons), and V_Pur_Restriction (vehicle purchase restriction) measure baseline vehicle demand. Among them, the V_Pur_Restriction restricts the purchase of fossil fuel motor vehicles, which would increase the relative attractiveness of NEVs to purchasers. The variable “Cold City” was used to control the impact of temperature on people’s willingness to buy NEVs. The charging efficiency of electric vehicles (EVs) is low, and the battery consumption is high in low temperature [55]. Therefore, in the cold regions, the functional requirements of NEVs, especially electric vehicles (EVs), are higher. The descriptive statistics of these control variables are shown in Table 3.

## 5. Results and Discussion

The results of the statistical models are shown in Table 4. We ran three regressions with the classic EHA model. We report the odds ratio, robust standard errors, Chi-square, McFadden R^2^ [56], Akaike information criterion (AIC) [57], and log-likelihood in Table 4. In Model 1, we only included the regional diffusion independent variable to test the classical hypothesis of regional diffusion. Model 2 adds all the independent variables, including regional diffusion, policy_sup_gov, guidance_sup_gov, administrative ranking, financial dependence, innovation capacity, and environmental pollution. Based on Model 2, we added a set of control variables in Model 3. From Models 1 to 3, we included three equally spaced natural cubic splines produced by BTSCS to test and control for temporal dependence. Because Model 3 is the most comprehensive, our interpretation of the results is mainly based on Model 3.

The first variable of interest is regional diffusion. The coefficient for this variable was statistically significant across all models, consistent with the regional diffusion hypothesis (H1), which predicts that a city’s decision to adopt a financial subsidy policy for NEVs is affected by the decisions of its neighbors. In Model 3, the odds ratio on regional diffusion was 1.037, indicating that for every 1 percent increase in the ratio of neighbors adopting the NEV policy, the odds of a city adopting NEV subsidy policy will increase by 3.7 percent, controlling for other variables. The adoption of NEV policy by other cities in the same province positively contributed to the adoption of local financial subsidy policy for NEVs in a city. This is consistent with the literature on policy diffusion, which provided consistent support for the regional diffusion hypothesis [34,35,36].

The top-down authoritarianism hypothesis is the second proposition that we were interested in testing in this study. In the Chinese top-down command-and-control administrative management system, the superior government has a great influence on the behavior of the lower-level governments. We expected that incentives and guidance mechanisms from a superior government positively contribute to the adoption of NEV local financial subsidies policy in city governments. In Models 2 and 3, the coefficient of “Policy_Sup_Gov” was significant at 0.05 level, and the odds ratios were both greater than 1, which strongly supports hypothesis H2a. But the coefficient for “Guidance_Sup_Gov” was not significant, providing no support for hypothesis H2b. The result indicates that the provincial government’s financial incentive positively contributed to the adoption of local financial subsidy policy for NEVs in city government, by increasing the odds of adopting of local financial subsidy policy for NEVs in a city by 78.9 present. However, the provincial government’s guidance mechanism did not have a positive impact on the city’s NEV policy adoption. In the current stage of development of NEVs in China, the financial incentive strategies of superior government, rather than the guidance strategies, will more effectively promote the diffusion of local financial subsidies for NEVs among cities. The possible reason is that the financial incentives of the provincial government can give city governments real benefits compared to the guiding mechanisms. Guiding mechanisms are more symbolic than financial incentives in their nature. Local governments would respond more positively in their actions to promote NEVs if the superior government makes a substantive financial commitment to NEVs.

The fourth variable we focused on was the administrative ranking. In our study, the city’s administrative ranking was divided into four categories from high to low, including centrally-administered municipality, sub-provincial-level city, provincial capital, and normal prefecture-level city. We expected that the city’s administrative ranking has a positive impact on the adoption of local financial subsidy policy for NEVs. In Models 2 and 3, the coefficient of administrative ranking was significant at 0.01 level, and the odds ratio was 3.113 in Model 3, lending strong support for the administrative ranking hypothesis (H3).

The fifth variable of interest was financial dependence, which measures a city’s financial situation. The local financial subsidy policy for NEVs requires that the city government use public finance to subsidize the purchasers of NEVs. Therefore, we expected cities with a good financial situation to be more likely to adopt policy. As shown in Table 4, the coefficient of financial dependence was statistically significant at the 0.01 level both in Models 2 and 3. In Model 3, the odds ratio of financial dependence was 0.965, indicating that for every additional 1 percentage point increase in financial dependence, the odds that the city adopted local financial subsidies for NEVs decreased by 3.5 present. That is consistent with the financial dependence hypothesis (H4).

In the innovation capacity hypothesis, we hypothesized the cities with stronger innovation capacity are more likely to adopt local financial subsidy policy for NEVs. Therefore, we expected the coefficient of innovation capacity to be positive. In Table 4, the coefficient was significant, and the odds ratio on innovation capacity was greater than 1, consistent with the innovation capacity hypothesis (H5). In Model 3, we found that the significance level of the coefficient of innovation capacity changed from 0.1 to 0.05, and the odds ratio also improved compared with Model 2. This indicates that innovation capacity has a much stronger effect on the adoption of NEV financial policy, after controlling for other variables.

The last variable of interest was environmental pollution. Current literature points out that an important motivation stimulating the development of NEVs is to alleviate serious environmental pollution problems [6,11,12,16,21]. Therefore, we expected that cities with more severe environmental pollution are more likely to adopt local financial subsidy policy for NEVs. However, the coefficient was statistically insignificant across all models, providing no support for the environmental pollution hypothesis (H6). This suggests that environmental pollution may not be a driving force for the adoption of NEV local financial subsidy policy. One possible reason is that the development of NEVs is at an early stage of development in China. Some scholars argued that vehicles equipped with engines will still account for more than 50% of the market in the next 30 years [30]. At present, compared with the vehicles equipped with engines, the market share of NEVs is very small, especially in small cities. Therefore, in most cities of China, the impact of NEVs on reducing environmental pollution is almost negligible.

Regarding the control variables, the coefficients on GDP growth rate, secondary industry, population, city road area, public_transp_vs, taxis, v_pur_restriction, and cold city were not statistically significant. The coefficient on GDP per capita was significant at the 0.05 level. However, the odd ratio on GDP per capita was almost equal to 1, so its impact on the adoption of NEV financial policy was minimal.

## 6. Conclusions

Designing and implementing effective NEV policy are policy priorities for policymakers and energy policy scholars. However, the formulation, adoption, and diffusion of the NEV policies have not been fully studied in the extant literature. This article explored the mechanisms driving the diffusion of local financial subsidy policy of NEVs in China. In this context, we aimed at analyzing the factors affecting the diffusion of local financial subsidies for NEVs in cities, to explain why some cities have taken the lead in adopting the NEV local financial subsidies policy, while other cities have lagged behind. Based on a data set of 286 cities in China from 2009 to 2016, and with the EHA method to analyze the strategic behaviors of local governments, we found that the number of city’s neighbors that had adopted the NEV policy, financial incentive policy of provincial government, administrative ranking of the city, city’s financial situation and innovation capacity had a direct impact on whether the city adopted local financial subsidy policy for NEVs. 

This study made empirical contributions to the extant literature and studies on NEVs. Most of the extant literature focuses on the impact of NEVs or the impact of government policies on the development of NEVs while ignoring the analysis of what factors affect the formulation and adoption of government’s NEV policy. Therefore, we rarely know the mechanism behind the government’s formulation, adoption, and diffusion of NEVs’ local financial subsidy policy. This study fills this gap and extends the literature on NEVs. At the same time, this study provides new ideas and directions for future research on NEV policies. Future research of NEV policies can code the policy adoption variable according to the value of NEV local financial subsidy to explore and examine the fundamental policy adoption mechanism. Moreover, researchers can compare the NEV local financial subsidies policy with other policies similar to the NEV policies, and verify whether they have similar mechanisms, which can provide guidance for broader policy practices.

The findings of this study have potential implications for policy practices. First, the empirical results suggest that financial condition is an important factor affecting the government’s adoption of NEVs. The financial incentives of the superior government and the financial situation of the local government both have a significant impact on policy adoption. The reason is that compared with relatively developed motor vehicles, NEVs are in the early stages of development with high initial investment cost, high uncertainty in technology and market, and inadequate infrastructure development. Therefore, it is necessary for the government to subsidize the NEVs and promote development of the NEVs through various policy instruments. Under the Chinese top-down command-and-control administrative management system, superior governments, such as the central government or provincial governments, can incentivize lower-level governments to formulate and implement local financial subsidy policy, such that the development plans of NEVs at the national and provincial levels can be translated into effective implementation. Second, local governments are irreplaceable policy actors in China. Each local government has unique characteristics. Some local governments with specific characteristics can play an important role in the adoption and diffusion of NEV policy. In this study, we found that cities with high administrative ranking, strong innovation capabilities, and good financial situation were more likely to adopt local financial subsidies for NEVs because they have the needs and ability to implement this policy. Therefore, the central governments can give priority to encouraging the implementation of the NEV local financial subsidies policy in cities with these characteristics, such as sub-provincial cities, provincial capital cities, and east coastal cities, to jump-start the development of NEVs in China. Under the mechanism of regional diffusion, other cities are likely to gradually adopt this policy through imitation and learning, and ultimately promote the spread of the NEVs nationwide. The mechanism which combined policy support of superior government and the leading role of cities with specific characteristics in policy innovation and adoption can effectively promote policy diffusion. This mechanism may be applicable to other policies similar to the financial subsidies for NEVs. However, some symbolic policies, such as the guidance mechanism of the superior government, cannot significantly prompt local governments to adopt policies. To urge local governments to respond more positively to the action of adopting new policies, it is necessary for superior governments to make a substantive commitment to policy support. This study differs from previous studies in highlighting the important role of top-down mechanisms in promoting the diffusion of policy innovation. The differentiation between substantive policy support versus symbolic policy guidance presents a unique view regarding the mechanism driving the diffusion of policy innovations at the local level.

## Figures and Tables

**Table 1:** Timeline of new policy adoption.

Year	Newly Adopted Cities	Number of Cities
2009	Shenzheng, Kunming	2
2010	Nanchang, Chengdu, Haikou	3
2011	Foshan, Jinan, Hangzhou, Tianjin, Zhengzhou, Zhuhai, Zhongshan, Beijing, Changchun, Jinhua, Hefei	11
2012	Wuhan, Xiangyang, Guangzhou, Dongguan, Shanghai	5
2013	Langfang, Jincheng, Nantong, Wuhu	4
2014	Zhuzhou, Longyan, Chongqing, Weifang, Ganzhou, Luzhou, Qingdao, Linyi, Fuzhou, Changsha, Huzhou, Suzhou, Dalian, Pingliang, Yangzhou, Ningbo, Yuncheng, Pingxiang, Xi’an, Nanjing, Jiujiang, Taiyuan, Yichun, Shaoxin, Huizhou, Xinzhou, Changzhou, Yancheng, Xinxiang, Shangrao, Mianyang, Nanning	32
2015	Xuchang, Fuzhou, Xiangtan, Xiamen, Taizhou, Quanzhou, Putian, Lanzhou, Ningde, Nanping, Fushun, Lianyungang, Xuzhou, Zhangzhou, Jiangmen, Jiayuguan, Xingtai, Linfen, Wulumuqi, Shijiazhuang, Handan, Baoding, Wuxi, Suqian, Liupanshui, Baotou, Shenyang, Zhengjiang, Chuzhou, Zhaoqing	30
2016	Zhoukou, Yiyan, Sanming, Shangqiu, Yantai, Zhumadian, Yanquan, Shantou, Jiaozuo, Taian, Liuzhou, Hebi, Luohe, Puyang, Anyang, Jinzhong, Qinyuan, Jieyang, Laibin, Datong, Jiuquan, Guiyang, Weinan, Zhongwei, Qingyang, Yan’an, Huhehaote, Tianshui, Yinchuan, Sanya, Lishui, Huaian, Tangshan, Suozhou, Luliang, Chizhou, Siping, Haerbin, Jiaxin, Taizhou, Anqing, Pingdingshan	42

**Table 2:** Variables, measures, and data sources.

Variables	Measures	Data Sources
**Dependent Variable**		
Adoption	1, if a city adopted the local financial subsidy policy in this year, otherwise, 0	Official web sites of city governments
**Independent Variables**		
Regional Diffusion	Accumulated percentage of city governments that have adopted local financial subsidy policy within each province by the same year	Calculated by authors
Policy_Sup_Gov	1, if a province issues financial subsidy policy in this year, otherwise, 0	Official Web sites of the provincial government
Guidance_Sup_Gov	1, if a province establishes a leading group or contact meeting system for promotion and application of new energy vehicles (NEVs) in this year, otherwise, 0	Official Web sites of the provincial government
Administrative Ranking	3: centrally-administered municipality; 2: sub-provincial-level city; 1: provincial capital but not a sub-provincial-level city; 0: normal prefecture-level city	China City Statistical Yearbook
Financial Dependence	Public finance expenditure minus public finance income, divided by public finance expenditure	China City Statistical Yearbook
Innovation Capacity	Quantity of invention patent granted by city divided by the total number of people (10,000 persons)	Patent cloud database
Environmental Pollution	Volume of Sulfur dioxide emissions	China City Statistical Yearbook
**Control Variables**		
GDP Growth Rate	Growth rate of real GDP	China City Statistical Yearbook
GDP per capita	GDP divided by total number of people	China City Statistical Yearbook
Secondary Industry	Percentage of GDP generated by secondary industry	China City Statistical Yearbook
Population	Household registered population at year-end (10,000 persons)	China City Statistical Yearbook
City Road Area	Area of city paved roads divided by total number of people	China City Statistical Yearbook
Public_Transp_Vs	Number of public transportation vehicles divided by total number of people (10,000 persons)	China City Statistical Yearbook
Taxis	Number of taxis divided by total number of people (10,000 persons)	China City Statistical Yearbook
V_Pur_Restriction	1, if a city has a vehicle purchase restriction policy; otherwise, 0	Official Web sites of city governments
Cold City	1, if the average temperature in the coldest month of city is below −10 °C; otherwise, 0	Provincial Statistical Yearbook

GDP, Gross domestic product.

**Table 3:** Descriptive statistics.

Variables	Mean	Std. Dev.	Min	Max	Obs
Adoption	0.062	0.242	0	1	2075
Regional Diffusion	10.61	15.954	0	92.31	2075
Policy_Sup_Gov	0.249	0.433	0	1	2075
Guidance_Sup_Gov	0.269	0.444	0	1	2075
Administrative Ranking	0.139	0.485	0	3	2075
Financial Dependence	54.376	21.867	−11.6	95.4	2075
Innovation Capacity	0.519	1.264	0	34.859	2075
Environmental Pollution	53,645.39	53,525.3	0.47	586,000	2075
GDP Growth Rate	10.839	4.218	−19.38	26	2075
GDP per capita	40,060.16	26,825.84	4491	257,000	2075
Secondary Industry	49.759	10.467	14.95	89.75	2075
Population	422.635	295.093	19.5	3375.2	2075
City Road Area	11.606	8.017	0.31	108.37	2075
Public_Transp_Vs	35.092	511.969	0.32	15281	2075
Taxis	21.561	18.157	1.35	184.05	2075
V_Pur_Restriction	0.006	0.076	0	1	2075
Cold City	0.165	0.372	0	1	2075

**Table 4:** Event history analyses of the diffusion of local financial subsidies policy for NEVs ^a.^

Variables	Model 1 (Odds Ratio)	Model 2 (Odds Ratio)	Model 3 (Odds Ratio)
Regional Diffusion	1.044 *** (0.005)	1.038 *** (0.007)	1.037 *** (0.007)
Policy_Sup_Gov		1.736 ** (0.475)	1.789 ** (0.503)
Guidance_Sup_Gov		1.243 (0.446)	1.115 (0.416)
Administrative Ranking		2.986 *** (0.626)	3.113 *** (0.835)
Financial Dependence		0.978 *** (0.006)	0.965 *** (0.009)
Innovation Capacity		1.155 * (0.087)	1.228 ** (0.123)
Environmental Pollution		1.000 (0.000)	1.000 (0.000)
GDP growth rate			0.998 (0.041)
GDP per capita			1.000 ** (0.000)
Secondary Industry			1.020 (0.016)
Population			1.001 (0.000)
City Road Area			1.012 (0.018)
Public_Transp_Vs			1.000 (0.000)
Taxis			1.006 (0.007)
V_Pur_Restriction			0.992 (0.808)
Cold city			0.830 (0.360)
_spline1	1.032 (0.107)	0.992 (0.116)	0.955 (0.115)
_spline2	0.915 (0.099)	0.939 (0.114)	0.963 (0.119)
_spline3	1.093 (0.083)	1.077 (0.091)	1.065 (0.091)
constant	0.014 *** (0.005)	0.011 *** (0.006)	0.009 *** (0.011)
Observations	2075	2075	2075
Chi-square	198.990	326.217	337.876
Pseudo r-squared	0.206	0.338	0.350
AIC	777.518	662.291	668.632
Log likelihood	−383.759	−320.146	−314.316

^a^ Note: *** *p* < 0.01, ** *p* < 0.05, * *p* < 0.1, Robust standard errors are given in parentheses. AIC, Akaike information criterion.
